# Can Monkeys Choose Optimally When Faced with Noisy Stimuli and Unequal Rewards?

**DOI:** 10.1371/journal.pcbi.1000284

**Published:** 2009-02-13

**Authors:** Samuel Feng, Philip Holmes, Alan Rorie, William T. Newsome

**Affiliations:** 1 Program in Applied and Computational Mathematics, Princeton University, Princeton, New Jersey, United States of America; 2 Department of Mechanical and Aerospace Engineering, Princeton University, Princeton, New Jersey, United States of America; 3 Howard Hughes Medical Institute and Department of Neurobiology, Stanford University, Stanford, California, United States of America; John Radcliffe Hospital, United Kingdom

## Abstract

We review the leaky competing accumulator model for two-alternative forced-choice decisions with cued responses, and propose extensions to account for the influence of unequal rewards. Assuming that stimulus information is integrated until the cue to respond arrives and that firing rates of stimulus-selective neurons remain well within physiological bounds, the model reduces to an Ornstein-Uhlenbeck (OU) process that yields explicit expressions for the psychometric function that describes accuracy. From these we compute strategies that optimize the rewards expected over blocks of trials administered with mixed difficulty and reward contingencies. The psychometric function is characterized by two parameters: its midpoint slope, which quantifies a subject's ability to extract signal from noise, and its shift, which measures the bias applied to account for unequal rewards. We fit these to data from two monkeys performing the moving dots task with mixed coherences and reward schedules. We find that their behaviors averaged over multiple sessions are close to optimal, with shifts erring in the direction of smaller penalties. We propose two methods for biasing the OU process to produce such shifts.

## Author Summary

Decisions are commonly based on multiple sources of information. In a forced choice task, for example, sensory information about the identity of a stimulus may be combined with prior information about the amount of reward associated with each choice. We employed a well-characterized motion discrimination task to examine how animals combine such sources of information and whether they weigh these components so as to harvest rewards optimally. Two monkeys discriminated the direction of motion in a family of noisy random dot stimuli. The animals were informed before each trial whether reward outcomes were equal or unequal for the two alternatives, and if unequal, which alternative promised the larger reward. Predictably, choices were biased toward the larger reward in the unequal reward conditions. We develop a decision-making model that describes the animals' sensitivities to the visual stimulus and permits us to calculate the choice bias that yields optimal reward harvesting. We find that the monkeys' performance is close to optimal; remarkably, the animals garner 98%+ of their maximum possible rewards. This study adds to the growing evidence that animal foraging behavior can approach optimality and provides a rigorous theoretical basis for understanding the computations underlying optimality in this and related tasks.

## Introduction

There is increasing evidence from *in vivo* recordings in monkeys that oculomotor decision making in the brain mimics a drift-diffusion (DD) process, with neural activity rising to a threshold before movement initiation [1]–[4]. In one well-studied task, monkeys are trained to decide the direction of motion of a field of randomly moving dots, a fraction of which move coherently in one of two possible target directions (T1 or T2), and to indicate their choice with a saccadic eye movement [5]–[7]. Varying the coherence level modulates the task difficulty, thereby influencing accuracy.

This paper addresses ongoing experiments on the motion discrimination task, but unlike most previous studies in which correct choices of either alternative are equally rewarded, the experiment is run under four conditions. Rewards may be high for both alternatives, low for both, high for T1 and low for T2, or low for T1 and high for T2. This design allows us to study the interaction between bottom-up (stimulus driven) and top-down (expectation driven) influences in a simple decision process. A second distinction with much previous work is that reponses are delivered following a cue, rather than given freely. We idealize this as an interrogation protocol (cf. [8]), in which accumulated information is assessed at the time of the cue rather than when it passes a threshold, and we model the accumulation by an Ornstein-Uhlenbeck (OU) process. Closely related work on human decision making is reported in [9],[10].

Consistent with random walk and diffusion processes [4], [11]–[15], neural activity in brain areas involved in preparing eye movements, including the lateral intraparietal area (LIP), frontal eye field and superior colliculus [7], [16]–[18], exhibits an accumulation over time of the motion evidence represented in the middle temporal area (MT) of extrastriate visual cortex. Under free response conditions, firing rates in area LIP reach a threshold level just prior to the saccade [19]. Further strengthening the connection, it has recently been shown that models of LIP using heterogeneous pools of spiking neurons can reproduce key features of this accumulation process [20],[21], and that the averaged activities of sub-populations selective for the target directions behave much like the two units of the leaky competing accumulator (LCA) model of Usher and McClelland [22]. In turn, under suitable constraints, the LCA can be reduced to a one-dimensional OU process: a generalization of the simpler DD process [8],[23],[24]. This allows us to obtain explicit expressions for psychometric functions (PMFs) that describe accuracy in terms of model and experimental parameters, and to predict how they should be shifted to maximize expected returns in case of unequal rewards.

The goals of this work are to show that PMFs derived from the OU model describe animal data well, that they can accommodate reward information and allow optimal performance to be predicted analytically, and finally, to compare animal behaviors with those predictions. Analyzing data from two monkeys, we find that, when faced with unequal rewards, both animals bias their PMFs in the appropriate directions, but by amounts larger than the optimal shifts. However, in doing so they respectively sacrifice less than 1% and 2% of their expected maximum rewards, for all coherence conditions, based on their signal-discrimination abilities (sensitivities), averaged over all session of trials. They achieve this in spite of significant variability from session to session, across which the parameters that describe their sensitivity to stimuli and reward biases show little correlation with the relationships that optimality theory predicts.

This paper extends a recent study that describes fits of behavioral data from monkeys learning the moving dots task, which also shows that DD and OU processes can provide good descriptions of psychometric functions (PMFs) [25]. A related study of humans and mice performing a task that requires time estimation [26] shows that those subjects also approached optimal behavior. The paper is organised as follows. After reviewing experimental procedures in the Methods section, we describe the LCA model and its reduction to OU and DD processes, propose simple models for the influence of biased rewards, and display examples of the resulting psychometric functions. The Results section contains the optimality analysis, followed by fits of the theory to data from two animals and assessments of their performances. A discussion closes the paper.

## Methods

### Behavioral Studies

To motivate the theoretical developments that follow, we start by briefly describing the experiment. More details will be provided, along with reports of electrophysiological data, in a subsequent publication.

#### Procedures

Two adult male rhesus monkeys, A and T (12 and 14 kg), were trained on a two-alternative, forced-choice, motion discrimination task with multiple reward contingences. Daily access to fluids was controlled during training and experimental periods to promote behavioral motivation. Prior to training, the monkeys were prepared surgically with a head-holding device [27] and a scleral search coil for monitoring eye position [28]. All surgical, behavioral, and animal care procedures complied with National Institutes of Health guidelines and were approved by the Stanford University Institutional Animal Care and Use Committee.

During both training and experimental sessions monkeys sat in a primate chair at a viewing distance of 57 cm from a color monitor, on which visual stimuli were presented under computer control. The monkeys' heads were positioned stably using the head-holding device, and eye position was monitored with a magnetic search coil apparatus (0.1° resolution; CNC Engineering, Seattle, WA). Behavioral control and data acquisition were managed by a PC-compatible computer running the QNX Software Systems (Ottawa, Canada) real-time operating system. The experimental paradigm was implemented in the NIH Rex programming environment [29]. Visual stimuli were generated by a second computer and displayed using the Cambridge Research Systems VSG (Kent, UK) graphics card and accompanying software. Liquid rewards were delivered via a gravity-fed juice tube placed near the animal's mouth, activated by a computer-controlled solenoid valve. Subsequent data analyses and computer simulations were performed using the Mathworks MATLAB (Natick, MA) programming environment.

#### Motion stimulus

The monkeys performed a two-alternative, forced-choice, motion discrimination task that has been used extensively to study both visual motion perception (e.g. [30]–[32]) and visually-based decision making [17],[33],[34]. The stimulus is composed of white dots, viewed through a circular aperture, on a dark computer screen. On each trial a variable proportion of the dots moved coherently in one of two opposite directions while the remaining dots flashed transiently at random locations and times (for details see [5]), and the animals reported which of two possible directions of motion was present. Discriminability was varied parametrically from trial to trial by adjusting the percentage of the dots in coherent motion: the task was easy if a large proportion of dots moved coherently (i.e. 50% or 100% coherence), but became progressively more difficult as coherence decreased. In what follows we indicate the motion direction by signing the coherence: thus +25% and −25% coherences are equally difficult to discriminate, but the coherent dots move in opposite directions. Typically, the animals viewed a range of signed coherences spanning psychophysical threshold. Animals were always rewarded for indicating the correct direction of motion, except that 0% coherence was rewarded randomly (50% probability) irrespective of their choices.

#### Experimental paradigm

**Figure 1:**
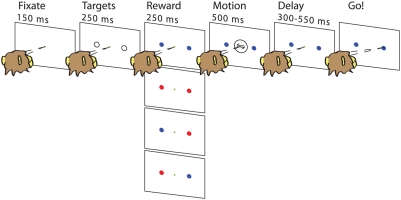
The motion discrimination task. Target colors cue the magnitude of rewards for correct responses, red denoting a value twice that of blue. The four panels in the reward segment show the possible reward conditions. See text for full description.

The horizontal row of panels in Figure 1 illustrates the sequence of events comprising a typical trial, which began with the onset of a small, yellow dot that the monkey must visually fixate for 150 msec. Next, two saccade targets appeared (open gray circles) 10° eccentric from the visual fixation point and 180° apart from each other, in-line with the axis of motion to be discriminated. By convention, target 1 (T1) corresponds to positive coherence and target 2 (T2) to negative coherence. After 250 msec the targets changed color, indicating the magnitude of reward available for correctly choosing that target. A blue target indicated a low magnitude (L) reward (1 unit, ≈0.12 ml of juice), while a red target indicated a high magnitude (H) reward (2 units). There were four reward conditions overall, schematized by the column of four panels in the Reward segment of Figure 1: (1) LL, in which both targets were blue, (2) HH, in which both were red, (3) HL, in which T1 was red and T2 blue, and (4) LH: the mirror image of HL.

The colored targets were visible for 250 msec prior to onset of the motion stimulus which appeared for 500 msec, centered on the fixation point. Following stimulus offset, the monkey was required to maintain fixation for a variable delay period (300–550 msec, varied across trials within each session), after which the fixation point disappeared, cueing the monkey to report his decision with a saccade to the target corresponding to the perceived direction of motion. The monkey was given a grace period of 1000 msec to respond. If he chose the correct direction, he received the reward indicated by the color of the chosen target. Fixation was enforced throughout the trial by requiring the monkey to maintain its eye position within an electronic window (1.25° radius) centered on the fixation point. Inappropriate breaks of fixation were punished by aborting the trial and enforcing a time-out period before onset of the next trial. Psychophysical decisions were identified by detecting the time of arrival of the monkey's eye in one of two electronic windows (1.25° radius) centered on the choice targets.

Trials were presented pseudo-randomly in block-randomized order. For monkey A, we employed 12 signed coherences, 0% coherence and four reward conditions, yielding 52 conditions overall. For monkey T we eliminated two of the lowest motion coherences because this animal's psychophysical thresholds were somewhat higher than those of monkey A, giving 36 conditions overall. We attempted to acquire 40 trials for each condition, enabling us to characterize a full psychometric function for each reward condition, but because the behavioral data were obtained simultaneously with electrophysiological recordings, we did not always acquire a full set for each condition (the experiment typically ended when single unit isolation was lost). For the data reported in this paper, the number of repetitions obtained for each experiment ranged from 19 to 40 with a mean of 36. The behavioral data analyzed here consists of 35 sessions from monkey A and 25 sessions from monkey T.

#### Behavioral training

Standard operant conditioning procedures were used to train both animals, following well-established procedures in the Newsome laboratory.

Monkey A began the study naive. His basic training stages were: (1) fixation task (3 weeks), (2) delayed saccade task (3 weeks), (3) direction discrimination task (3 months), and (4) discrimination task with varied reward contingencies (2 months). Training on motion discrimination began with high coherences only and a short, fixed delay period. White saccade targets cued small, equal rewards. As the animal's psychophysical performance improved, we progressively added more difficult coherences. When the range of coherences fully spanned psychophysical threshold, we slowly extended the duration and variability of the delay period to the final desired range. At this stage the monkey was performing the final version of the task, lacking only the colored reward cues. After establishing stable stimulus control of behavior in this manner, we introduced all four reward contingencies simultaneously. Following a brief period of perseveration on the H reward condition, Monkey A learned reasonably quickly to base decisions on a mixture of motion and reward information. Training continued until psychophysical thresholds and bias magnitude stabilized.

Monkey T had performed the basic direction discrimination task for a period of years before entering this study. We therefore began by shaping this animal to perform the discrimination task with the same timing as for monkey A (2–3 weeks). Once his performance stabilized, we again introduced the four reward conditions simultaneously. This animal took much longer than monkey A to adapt to the new reward contingencies: about five months. He seemed to explore a wider range of erroneous strategies before settling on the correct one. While it is tempting to attribute this to his earlier extended performance of the task with equal reward contingencies, we do not know this to be true. Regardless, the behavioral endpoints were very similar for the two animals, and we therefore conclude that the different training histories were not relevant to the results of this study. We did not explicitly shape the magnitude or direction of the behavioral bias for either monkey; we simply trained the animals until threshold and bias became asymptotic. Target colors (red and blue) and associated reward magnitudes (H and L) were fixed throughout the entire run of training and experimental sessions.

### Models for Evidence Accumulation and Choice

We now describe a simple model for two-alternative forced-choice (2AFC) tasks. Several other models are reviewed in [8], along with the relations among them and conditions under which they can be reduced to OU and DD processes. The model yields explicit expressions that predict psychometric functions and that reveal how these functions depend upon parameters describing the stimulus discriminability and reward priors. While optimality analyses can be conducted using fitted PMFs such as sigmoidal functions, our derivation links the behavioral data to underlying neural mechanisms.

#### The leaky competing accumulator model

The LCA is a stochastic differential equation [35] whose states 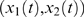 describe the activities of two mutually-inhibiting neural populations, each of which receives noisy sensory input from the stimulus, and also, in the instantiation developed here, input derived from reward expectations. See [22],[36]. The system may be written as
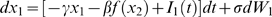
(1)

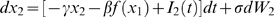
(2)where 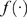 is a sigmoidal-type activation (or input-output) function, 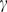 and 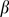, respectively, denote the strengths of leak and inhibition, and 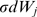 are independent white noise (Weiner) increments of r.m.s. strength *σ*. The inputs 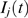 are in general time-dependent, since stimulus and expectation effects can vary over the course of a trial. To fix ideas, we may suppose that the states 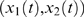 represent short-term averaged firing rates of LIP neurons sensitive to alternatives 1 and 2. We recognize that the decision may be formed by interactions among several oculomotor areas, but note that a partial causal role for LIP has been demonstrated [34].

Under the interrogation protocol the choice is determined by the difference 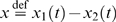: if 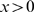, T1 is chosen, and if 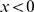, T2 is chosen. As explained in [8], this models the “hard limit” of a cued response, in which subjects may not answer before the cue, and must answer within a short window following it, to qualify for a reward.

#### Reduction to an Ornstein-Uhlenbeck process

**Figure 2:**
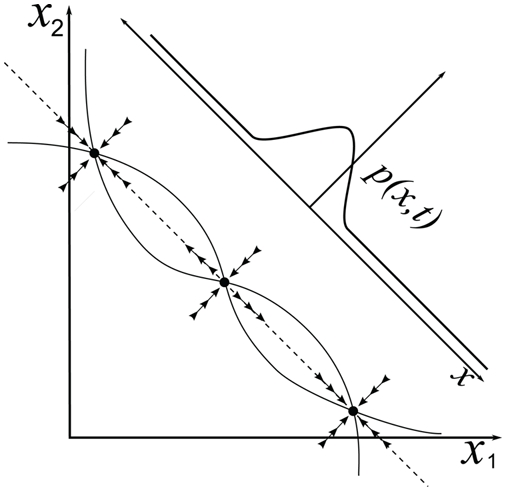
A typical state space of the LCA model, showing nullclines on which 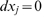 for 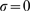 (thin curves), fixed points (filled circles with arrows indicating stability types) and slow manifold (dashed line). Diagonal solid line represents one-dimensional state space 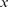 of reduced OU model, with associated probability distribution 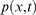 of sample paths.

In the absence of noise (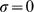) and with *constant* inputs 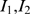, equilibrium solutions of Eqs. (1–2) lie at the intersections of the nullclines given by 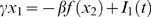 and 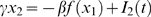, and, depending on the values of the parameters 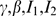 and the precise form of 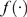, there may be one, two or three stable equilibria, corresponding to low activity in both populations, high activity in 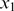 and low in 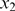, and vice-versa. If the nullclines lie sufficiently close to each other over the activity range that encompasses the equilibria, it follows that a one-dimensional, attracting, slow manifold exists that contains both stable and unstable equilibria, and solutions that connect them [23],[37]: see Figure 2. With 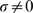 (and 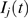 non-constant), we must appeal to the theory of stochastic center manifolds to draw a similar, probabilistic conclusion ([38],[39] and Chapter 7 of [40]). For reduction of higher-dimensional and nonlinear neural systems, see [41].

To illustrate, we simplify Eqs. (1–2) by linearizing the sigmoidal function at the central equilibrium point 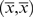 in the case of equal inputs *I_j_*(*t*)≡*I*, where 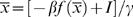. Parameterizing the sigmoid so that 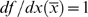, Eqs. (1–2) become
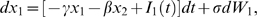
(3)

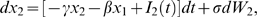
(4)and subtracting these equations yields a single scalar SDE for the activity difference *x*:
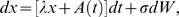
(5)where 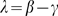, 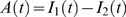 and 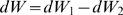 are independent white noise increments. Thus, if stimulus A is displayed, we expect 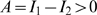 and vice versa.

Eq. (5) describes an OU process, or, for 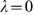, a DD process. The DD process is a continuum limit of the sequential probability ratio test [8], which is optimal for 2AFC tasks in that it delivers a decision of guaranteed accuracy in the shortest possible time, or that, given a fixed decision time, it maximizes accuracy [42],[43]. The latter case is relevant to the cued responses considered here.

#### Prediction of psychometric functions

The probability of choosing alternative 1 under the interrogation protocol can be computed from the probability distribution of solutions 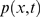 of Eq. (5), which is governed by the forward Kolmogorov or Fokker-Planck equation [44]:
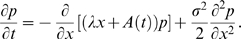
(6)When the distribution of initial data is a Gaussian (normal) centered about 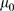,
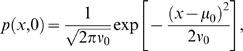
(7)solutions of (6) remain Gaussian as time evolves:
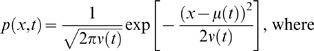
(8)

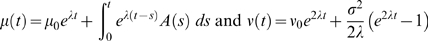
(9)contain integrated stimulus and noise respectively. Note that 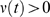 regardless of the sign of 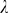, so the square root in Eq. (11) is well-defined. In the DD limit 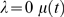 and 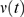 simplify to
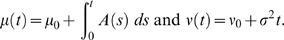
(10)


Henceforth we set 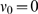, assuming that all sample paths start from the same initial condition 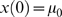. From Eq. (10) the probability that T1 is chosen at time 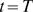 can be computed explicitly as a cumulative normal distribution:
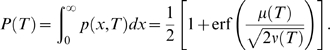
(11)Here 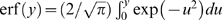 denotes the error function and Eq. (11) represents a *psychometric function* (PMF) whose values rise from 0 to 1 as the argument 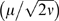 runs from −∞ to +∞, so multiplying it by 100 gives the expected percentage of T1 choices.

In addition to its dependence on viewing time 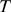, the PMF also depends on the functional forms of the drift and noise terms embedded in 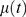 and 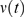. In particular 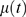 depends on the coherence or stimulus strength via 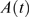, and upon prior expectations or biases that reward information might introduce, for example via 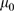 (examples are provided in the next subsection). To emphasize this we sometimes write the PMF as 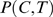 or 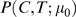, to denote its dependence on 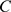 and other parameters. Specifically, we shall examine two aspects of the PMF as a function of 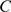: the *slope* 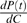 at 50% accuracy, and the *shift*: the value of 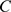 at which 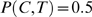, or equivalently, where 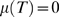.

#### Models of stimuli and reward biasing

Following [45],[46], we suppose that the part of the drift rate due to the stimulus depends linearly on coherence: *A*_stim_ = *aC*. (While power-law dependence on 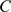 has been introduced to account for behavior early in training, a linear relationship seems generally adequate for well-trained animals [46].) Here 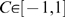 (between 100% leftward and 100% rightward motion coherence), as determined by the experimenter, and 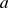 is a scaling or sensitivity parameter that allows one to fit data from different subjects, or from one subject during different epochs of training (Figure 14 of [25]).

We propose two strategies to account for prior reward information. The first and simplest is to bias the initial condition at stimulus onset 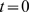, taking 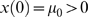 if T1 garners a higher reward (HL) and 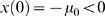 if T2 does so (LH), with 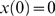 for equal rewards (LL and HH). In this case, from Eq. (9), the integrated drift rate and noise levels are:
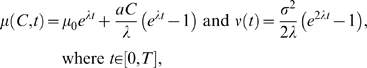
(12)and the decision is rendered at the end of the motion period 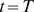. Such biasing of initial data is optimal for the free response protocol if coherences remain fixed over each block of trials [8], but, as we shall see, other strategies can do equally well under the interrogation protocol.

Alternatively, motivated by the task sequence of Figure 1, and as suggested by J.L. McClelland (personal communication), one can assume that bias enters throughout a reward indication period (marked “targets” in Figure 1) of duration 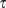 *and* the ensuing motion period, as a drift term upon upon which the stimulus is additively superimposed to form a piecewise-constant drift rate:
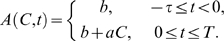
(13)From Eqs. (9) the resulting integrated drift and noise during the motion period 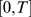 are
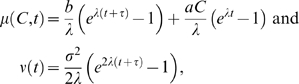
(14)where we set 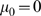, since 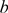 accounts for reward bias, with 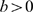 if T1 has higher reward, 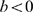 if T2 has higher reward and 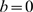 for equal rewards. Note that accumulation of reward information now begins at 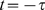.

The first model assumes that reward information is assimilated during the target period 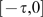 and loaded into the initial accumulator state 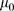 at motion onset 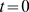, after which it is effectively displaced by the stimulus. In the second strategy the reward information 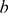 continues to apply pressure throughout the motion period 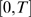. (Presumably 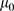 and 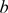 should scale monotonically, but not necessarily linearly, with reward ratio.) These represent extremes of a range of possible strategies. More complex time-varying drift functions could be proposed to model reward expectations, waxing and waning attention to stimuli, and for the fixation, target and delay periods, but analyses of electrophysiological data (LIP firing rates), currently in progress, are required to inform such detailed modeling. Here we simply assume that the accumulation process starts at reward cue onset (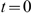 or 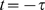) and ends at motion offset (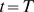), the decision state being preserved until the cue to respond appears. Moreover, as we now show, lacking data with variable stimulus and/or reward information times, it is impossible to distinguish between models even as simple as the two described above.

The PMF (11) depends only upon the ratio 
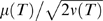
 (which is one half the descriminability factor *d*′ of Eq. (7) of [22], cf. [47]), and in Eqs. (12) and (14) reward biases appear as additive factors in the numerator 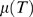. Thus, if all parameters other than 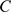 are fixed, and 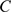 appears linearly as assumed above, the argument of the PMF can be written in both cases in the simple form 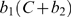, so that
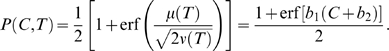
(15)Here 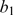 and 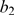 respectively determine the slope and shift of the PMF: the slope at 50% T1 choices being 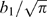 in the units of probability of a T1 choice per % coherence, and 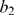 having the units of % coherence. In turn, 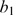 and 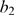 depend upon the parameters 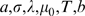, and 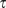 introduced above; for the specific cases of Eqs. (12) and (14), we respectively have:
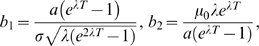
(16)

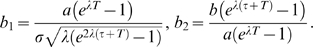
(17)


The ratios 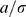 and 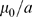 or 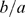 in Eqs. (16) and (17) characterize a subject's ability to extract information from the noisy stimulus, and the weight placed on reward information relative to stimulus. Experiments in which 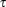 and 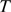 are varied independently could in principle distinguish between these cases, but with the present data we can only fit the slope 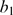 and shift 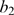. Nor can we determine whether the process is best described by a pure DD process with 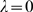 and constant drift 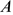, or an OU process with 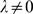, or, indeed, whether the drift rate varies with time. Recent experiments on human subjects with biased rewards that use a range of interrogation times [9],[10] suggests that a leaky competing accumulator model [22] is indeed appropriate, and data from those experiments may allow such distinctions to be made.

#### Examples of psychometric functions

**Figure 3:**
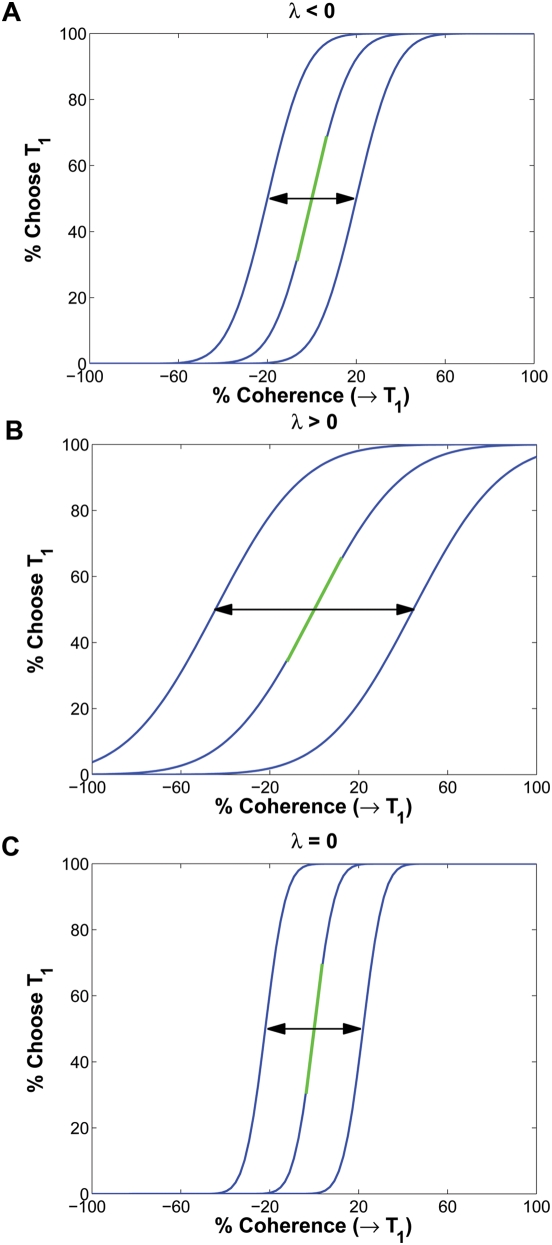
Psychometric functions showing fraction of T1 choices as a function of coherence 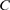 for constant reward bias 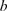 applied before and during motion period. (A) 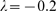; (B) 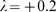; (C) 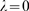; each panel shows the cases 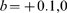 and −0.1 (left to right). Remaining parameters are 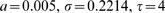 and 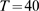 (arbitrary time units). Green lines indicate slopes for zero bias; arrows show shifts.

To illustrate how PMFs depend upon the parameters describing evidence accumulation (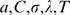) and reward biasing (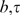), we compute examples based on the second model described above. Substituting the expressions (14) in Eq. (11), we obtain:
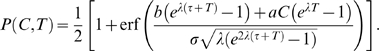
(18)In case 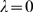 the exponential expressions simplify (cf. Eqs. (10)), giving:
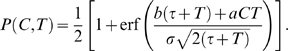
(19)Examples of these PMFs are plotted in Figure 3 for 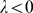, 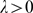 and 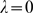. Parameter values, listed in the caption, are chosen to illustrate qualitative trends. Note that the slopes of the functions are lower for 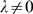 (top row) than for 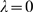 (bottom), and lowest for 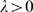 (middle), illustrating that the DD process 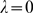 is optimal. Also, for fixed 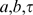 and 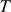, the PMFs are shifted to the left or right for 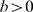 and 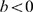 respectively, by an amount that grows as 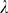 increases from negative to positive.

To understand these trends, we recall that a stable OU process (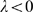) exhibits recency effects while an unstable one (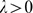) exhibits primacy effects [22]. In the former case information arriving early decays, while for 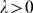 it grows, so that reward information in the pre-stimulus cue period exerts a greater influence, leading to greater shifts. Unstable OU processes also yield lower accuracy than stable processes. Specifically, the factor 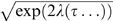 in Eq. (18) reflects the fact that noise accumulates during the cue period, leading to accelerating growth of solutions when 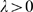 which the stimulus cannot repair. In general, while accuracy increases monotonically with viewing time, it approaches a limit below 100% for any 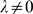: specifically:
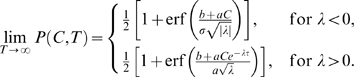
(20)


The slopes of the PMF can clearly be increased by setting 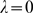 and raising the sensitivity-to-noise ratio 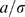, but these parameters are constrained for individual subjects by physiological factors and by training. Indeed, Eckhoff et al. [25] find that 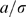 and 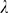 remain stable over relatively long periods (several sessions) for trained animals. As noted below Eqns. (15–17), the present data does not allow us to estimate such “detailed” parameters. In the analysis to follow we therefore adopt the two-parameter form of Eq. (15), regarding the PMF slope 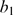, which quantifies sensitivity to stimulus, as fixed, and seeking shifts in 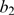 that maximize the overall expected reward for that sensitivity, although this implies a causal chain that animals may not follow, as we note in the Discussion.

## Results

### Optimality Analysis

Given a fixed slope 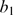, we now ask what is the shift 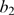 in the PMF that maximizes expected rewards in the case that the two alternatives are unequally rewarded. How much should the subject weight the reward information relative to that in the stimulus, in order to make optimal use of both?

#### Two motivating examples

Let 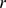 denote the reward obtained on a typical trial, namely, 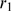 if alternative 1 is offered and chosen, and 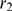 if 2 is offered and chosen. The *expected reward* 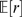 is obtained by multiplying each 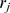 by the probability that the corresponding alternative is chosen, when it appears in the stimulus. To make this explicit, first suppose that coherence is fixed from trial to trial and that the two possible stimuli 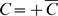 (T1) and 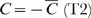 (T2) are equally likely. In this case
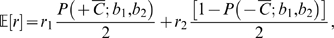
(21)where we use the fact that 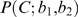 and 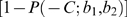 are the average proportions of correct T1 choices and T2 choices for coherences 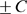 and we write the argument of 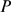 explicitly to indicate its dependence on coherence and the slope and bias parameters introduced in Eq. (15).

Using Eq. (15) and the fact that
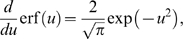
(22)we may compute the derivatives of 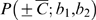 with respect to 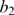 to derive a necessary condition for a maximum in 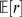:
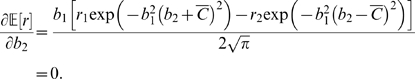
(23)This implies that
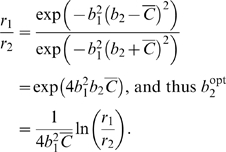
(24)To verify that (24) identifies the global maximum we compute the second derivative at 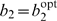:
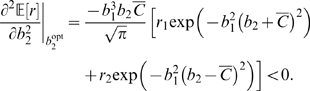
(25)


**Figure 4:**
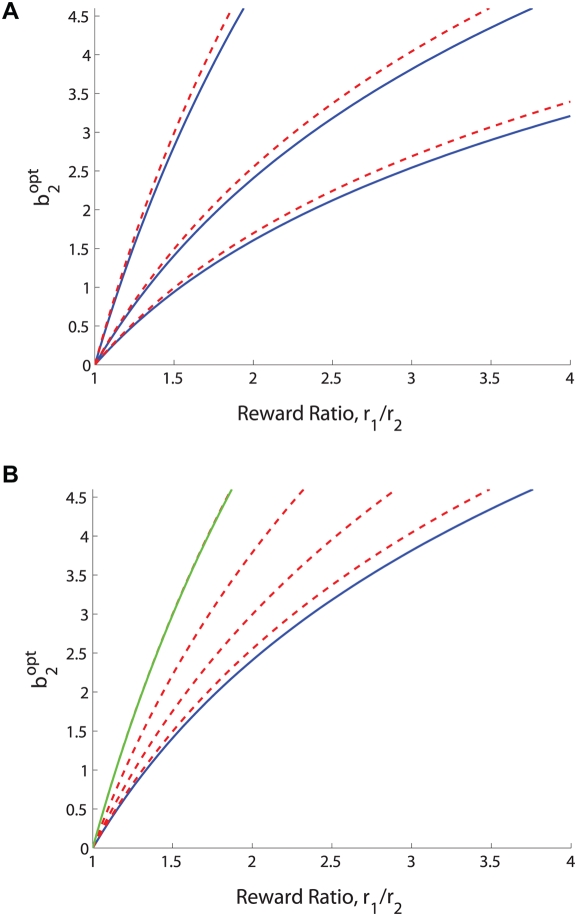
Optimal shifts 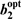 as a function of the reward ratio *r*_1_/*r*_2_ for fixed coherences (solid blue curves) and for coherence ranges centered on the fixed coherences (dashed red curves). (A): 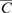 = 10; 20 and 30% (top left to bottom right, solid blue), and [*C*_1_;*C*_2_] = [5; 15]; [15; 25] and [25; 35] (top left to bottom right, dashed red). (B): Coherence bands centered on 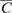 = 20% (solid blue curve) with widths 10; 20; 30 and 40% (bottom left to top right, dashed red). Approximation of Eq. (30) shown in green. The slope *b*_1_ is fixed at 0.06 throughout.

For equal rewards 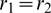 we recover 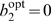: an unbiased PMF with 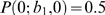, and for a fixed reward ratio, 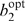 varies inversely with 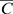, approaching ∞ as 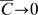. In this limit the stimulus contains no information and it is best to always choose the more lavishly rewarded alternative. Figure 4A (top panel, solid blue curves) shows examples of 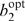 plotted as a function of reward ratio for fixed 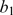 and three different coherence levels.

Coherences are mixed during blocks of trials in the experiment of interest, so we now consider a continuum idealization in which coherences are selected from a uniform distribution over 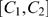 (again positive for T1 and negative for T2). Instead of summing the weighted probabilites of correct 1 and 2 choices for 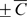, we must now average over the entire range of coherences:
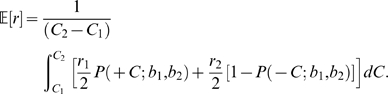
(26)Computing the derivative via the Leibniz integral rule, noting that the limits of integration do not depend on 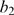, and again using Eq. (22) we find that
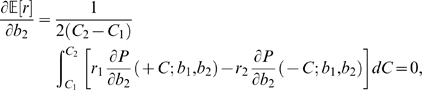
which implies that
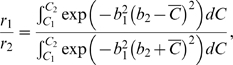
(27)where we have cancelled common terms in the integrands that do not depend upon 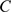. To turn these expressions into standard error function integrals we change variables by setting 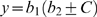 and 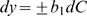. Integrating Eq. (27) and cancelling further common terms yields the optimality condition:
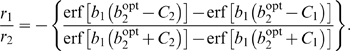
(28)Setting 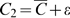, 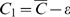, expanding (28) in a Taylor series and letting 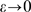, we recover the single coherence level result (24).

The expression (28) cannot be inverted to solve explicitly for the optimal starting point 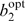 in terms of the the other parameters, but we may use it to plot the reward ratio 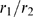 as a function of 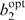 for fixed 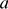, 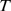, 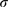 and coherence range 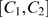. The axes of the resulting graph can then be exchanged to produce a plot of 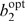 vs. 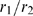 for comparison with the single coherence prediction (24). The dashed red curves in Figure 4A show optimal shifts for 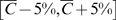 centered around the three fixed coherence levels (solid blue curves). Figure 4B shows optimal shifts for coherence bands of increasing width centered around 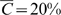. Note that the coherence bands require larger biases than fixed coherences at their centers demand (top panel), and that optimal bias increases with the width of a band centered on a given coherence (bottom panel). Biases, and hence optimal shifts of the PMF, increase with coherence range because the reward information is more significant for coherences close to zero, where accuracy is lowest. This fact will play a subtle role when we compare optimal shifts predicted for the two monkeys, one of which worked with a smaller set of coherences than the other.

If coherences span the range from 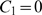 to an upper limit 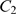 that is sufficently large that we may approximate
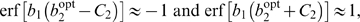
(29)then (28) implies that
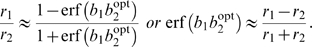
(30)(Note that 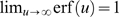 and 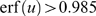 for 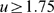, and that the latter condition holds for the parameters estimated for both monkeys below.) Eq. (30) in turn implies that, instead of the relationship 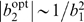 of Eq. (24) in the single coherence case, for a sufficiently broad band of coherences including zero, we have 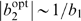 or 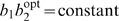. The green curve in Figure 4B shows that this simple relationship can provide an excellent approximation.

#### Optimal shifts for a finite set of coherences

In the present experiment a finite set of fixed nonzero coherences 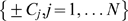 is used, along with zero coherence, each of these 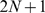 conditions being presented with equal probability. Moreover, zero coherence stimuli (for which there is no correct answer) are rewarded equally probably with 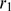 and 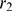. The expected reward on each trial is therefore:
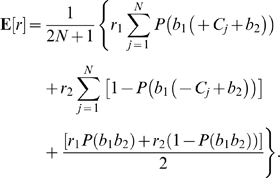
(31)As in the preceding subsection the optimal shift is determined by seeking zeros of the derivative of (31) with respect to 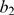. Excluding the normalization factor 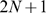, this leads to:
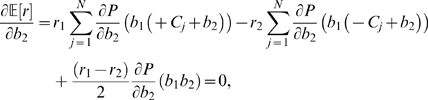
(32)from which, again appealing to Eq. (22), we obtain the expression
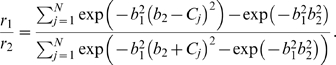
(33)As for Eq. (28) we cannot solve Eq. (33) explicitly for 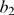 in terms of the reward ratio and 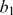, but we can again plot 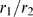 as a function of 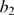 for fixed 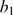 values, and invert the resulting graph, as is done in Figure 6 below.

**Figure 5:**
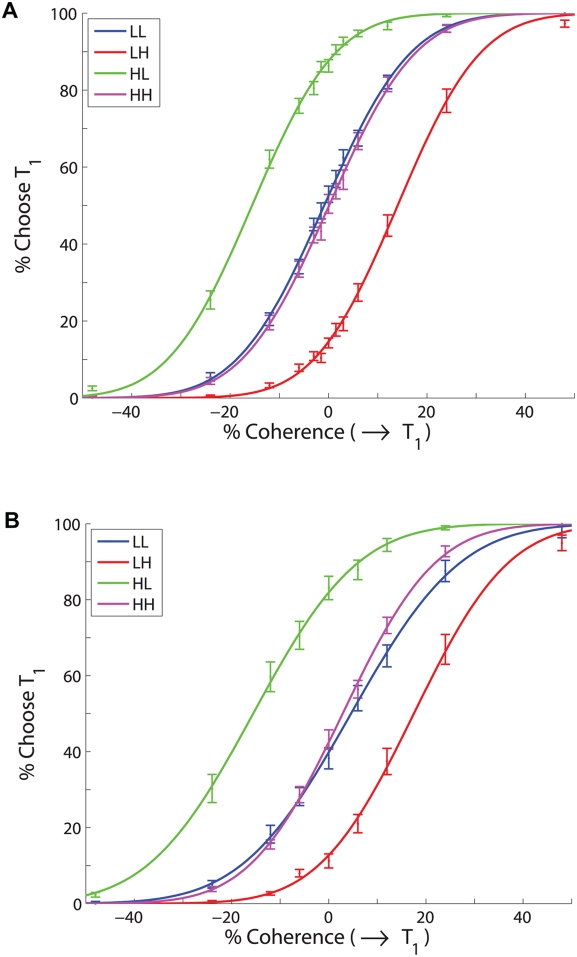
Fits of accuracy data from monkeys A (A) and T (B) to the PMF (15), for the four reward conditions averaged over all sessions. Bars denote standard errors. See text for details.

To get an explicit idea of how the key quantities of slope 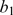, shift 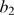 and reward ratio 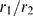 are related at optimal performance, we recall the relationships (24) and (30) derived for the special cases of a single coherence and a broad range of uniformly-distributed coherences including zero. These predict, respectively, that 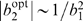 and 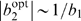. For non-uniformly distributed coherences such as those used in the present experiments, we have found that a function of the form
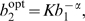
(34)with 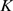 and 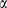 suitably chosen constants that depend upon the set of coherences and the reward ratio, fits the optimal shift-sensitivity relationship very well; we shall appeal to this in analyzing some of the experimental data in the next section. In all cases, optimal shifts increase rapidly as sensitivity (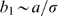) diminishes.

### Fitting the Theory to Monkey Data

Here we perform fits of accuracy data collected for a discrete set of coherences, namely 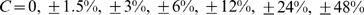, under the four reward schedules described under Experimental paradigm. As noted there, T was not tested with the lowest coherences 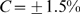 and ±3%. Data from the two monkeys (A and T) are analyzed separately. While each coherence is presented with equal probability, their spacing increases with 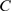, so that the majority of trials occurs in the center of the range around 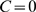, unlike the case of uniformly-distributed coherences. This will play a subtle role when we compare optimal shifts for the two animals.

#### Fits of data averaged over multiple sessions to PMFs

Drawing on the observations in Models of stimuli and reward biasing, we start by estimating average values of the parameters 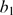 and 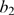 in the psychometric function in the form (15), by collectively fitting all the data for each animal: 35 blocks of trials for A and 25 for T. We first fitted 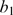 and 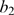 separately for the four reward conditions by computing the fraction of T1 choices 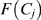 for each coherence level and minimizing the residual error:
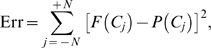
obtaining the values in the top two rows of Table 1. Fits were done using MATLAB's lsqnonlin with default options (Matlab codes used for data analysis, computation of statistics, and producing figures are available at www.math.princeton.edu/˜sffeng). Figure 5 shows the resulting PMFs for A (top) and T (bottom). We then pooled the accuracy data for equal rewards, re-fitted to determine common 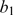 and 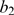 values for conditions HH and LL for each animal, and held 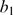 at the resulting value while re-estimating 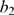 for the unequal rewards data, to obtain rows 3 and 4 of the table. The bottom two rows list values of 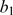 and 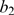 obtained when 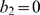 is imposed in separate fits of conditions LL and HH (first two columns), and the value of 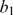 obtained from pooled HH and LL data with 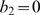, along with values of 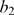 for unequal rewards obtained using that same 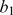 value (last two columns). Fit errors are substantially higher for monkey T under the 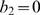 constraint, due to his greater shifts for LL and HH (figures in parentheses in last row). PMFs obtained using the 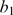 and 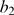 values from the lower four rows of Table 1 are very similar to those of Figure 5 (not shown).

**Figure 6:**
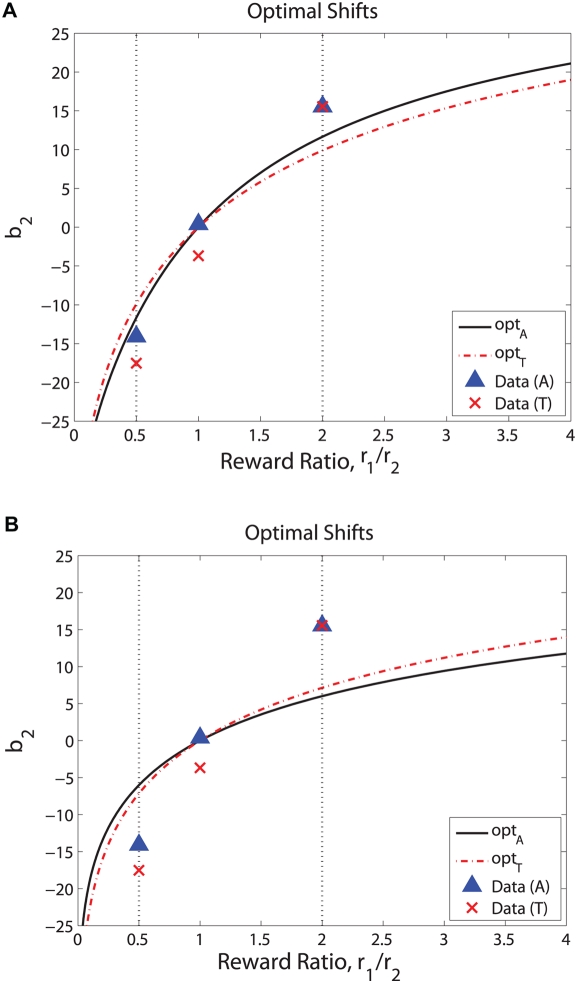
Optimal shifts *b*_2_ for a range of reward ratios *r*_1_/*r*_2_ and *b*_1_ = 0.0508 (solid, black) and *b*_1_ = 0.0432 (dot-dashed, red), corresponding to slopes of PMFs fitted to equal rewards data for monkeys A and T. Vertical dotted lines at *r*_1_/*r*_2_ = 0.5 and 2 intersect the curves at the symmetrically-placed optimal shifts for those reward ratios. (A) Predictions for the different sets of nonuniformly-distributed coherences viewed by each animal. (B) Results for coherences distributed uniformly from −48% to 48%: note smaller optimal shifts and reversal of order of curves for A and T compared to panel A. Triangles and crosses respectively indicate shifts determined from data for monkeys A and T for *r*_1_/*r*_2_ = 0.5, 1 and 2 (cf. Table 1).

**Table 1:** Parameter values for data fits for monkeys A and T, averaged over all sessions, to the PMF (15).

Subject	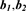 for LL	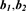 for HH	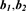 for HL	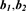 for LH
Monkey A	0.0509, 0.890 (0.00096)	0.0509, −0.110 (0.0011)	0.0526, 15.5 (0.0017)	0.0531, −14.0 (0.0013)
Monkey T	0.0399, −4.58 (0.00087)	0.0469, −2.87 (0.00057)	0.0415, 15.6 (0.00081)	0.0460, −17.5 (0.0018)
Monkey A	0.0508, 0.390 (0.00036)	0.0508, 0.390 (0.00036)	0.0508, 15.8 (0.0020)	0.0508, −14.3 (0.0018)
Monkey T	0.0432, −3.68 (0.00023)	0.0432, −3.68 (0.00023)	0.0432, 15.4 (0.0011)	0.0432, −17.9 (0.0024)
Monkey A	0.0507, 0 (0.0059)	0.0509, 0 (0.0012)	0.0508, 15.8 (0.0013,0.0020)	0.0508, −14.3 (0.0013,0.0018)
Monkey T	0.0385, 0 (0.047)	0.0460, 0 (0.023)	0.0421, 15.5 (0.033,0.00085)	0.0421, −18.0 (0.033,0.0030)

Upper two rows show separate fits of 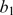 and 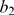 for the four reward conditions. Middle two rows show fits for pooled LL and HH data, with resulting common 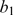 value held fixed across unequal reward conditions. Lower two rows show results with 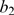 constrained to zero for equal rewards; in columns 1 and 2 LL and HH are fitted separately, in columns 3 and 4 LL and HH data is pooled to produce 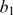, and this value is fixed across unequal reward conditions. Units of 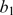 and 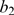 respectively are increase in probability of a T1 choice per change in % coherence, and % coherence (see Models of stimuli and reward biasing). Values are given to 3 significant figures with residual fit errors (in mean square norm) in parentheses. In rows 5 and 6 of the HL and LH columns the first error figure refers to the LL and HH pooled data fit with 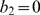.

In the first and least-constrained fits, Monkey A's 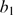 values change across the four reward conditions by a factor of only 1.05, indicating that the predominant effect of unequal rewards is a lateral shift of the PMF, with no significant change in slope. His shifts for the HL and LH conditions are significantly different from zero and from those for HH and LL (according to one- and two-sample t tests on the underlying normal distributions 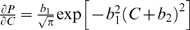 with parameters listed in the top row of Table 1 and 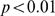 (section 9.2 of [48])). At 15.5% and −14.0% the HL and LH shifts are not significantly asymmetrical (t test, 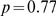), and his PMFs for equal rewards are also statistically indistinguishable from each other (t test, 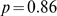) and from an unshifted PMF with 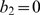 (t tests, 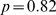). In contrast, Monkey T displays slopes that differ by a factor of 1.18 and shifts toward T2 of 4.58% and 2.87% respectively in the the LL and HH conditions, his slope being lower and his shift larger for LL than for HH, possibly indicating increased attention in the case of high rewards. However, his PMFs for LL and HH are also statistically indistinguishable (t test, 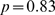) and, in spite of the more obvious asymmetry their shifts are also not significantly different from zero (t tests, 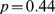). Like A's, his PMFs for the unequally rewarded conditions are significantly shifted (t tests, 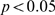), but again without significant asymmetry (t test, 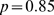).

In the optimality analysis to follow we require a common estimate of slope as a measure of the animal's sensitivity, or ability to discriminate the signal. Rows 3 and 4 of Table 1 show that shifts for the unequally rewarded conditions change by at most 0.4% when 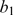 is held at the common value fitted to the equal rewards data. We therefore believe that the common slope estimates 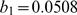 for monkey A and 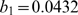 for monkey T are suitable bases for optimality predictions. We have already noted that monkey T's higher psychophysical threshold led us to exclude the ±1.5% and ±3% coherences, and his common slope value is substantially less than that of monkey A.

Finally, we computed rows 5 and 6 of Table 1 with 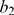 constrained to zero in order to check that the slope parameter is not significantly affected by shifts and left/right asymmetries in the equally rewarded cases. Monkey A's slope is unchanged (to 3 significant figures) and Monkey T's distinct LL and HH slopes change by factors of only 0.96 and 0.98. Even when a common fit to LL and HH data with 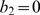 is enforced, Monkey T's shifts for unequal rewards change by only 0.1%, and monkey A's are unchanged.

We remark that the sigmoidal or logit function
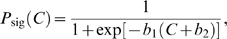
(35)used in the work reported in [9],[10], provides an alternative model for the PMF. We examined fits to 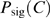 and found that they were generally similar to the cumulative normal fits, but typically incurred slightly higher residual fit errors. Eq. (35) appears simpler than the cumulative normal distribution (15), which involves the error function, but after taking derivatives to compute optimal shifts, the final conditions are no easier to use. More critically, Eq. (35) lacks a principled derivation from a choice model.

#### How close are the animals, on average, to optimal performance?

We took the slope values 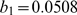 for A and 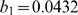 for T, fitted to the pooled LL and HH equal rewards data averaged over all sessions (rows 3 and 4 of Table 1) to best represent the animals' average sensitivities. Using these values, we then computed optimal shifts predicted by Eq. (33) for unequal reward conditions over the range 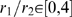, which includes the ratios 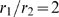 (HL) and 0.5 (LH) that were tested. We did this both for the sets of coherences viewed by A and T, and for a uniformly distributed set of coherences spanning the same range. Figure 6 shows the resulting optimal shift curves along with the actual session-averaged shifts computed from the animals' unequal reward data as listed in the top two rows of Table 1, and the common values for equal rewards as listed in rows 3 and 4 (triangles and crosses). Both animals “overshift” beyond the optimal values for the LH and HL conditions, T's overshifts being greater than A's. The figure also clearly shows T's appreciable shift for equal rewards, in contrast to A's nearly optimal behavior under those conditions.

Figure 6A shows that, when based on the coherences used in the experiment, monkey T's optimal curve predicts shifts *smaller* than those for monkey A, despite T's lower sensitivity. For a given reward ratio and the *same* set of coherences, a smaller 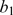 requires *greater* shifts because, as sensitivity falls, it is better to place increasing weight on the alternative that gains higher rewards, as shown in Figure 6B. However, since monkey A views four low coherence stimuli that T does not (±1.5% and ±3%), his optimal shifts are additionally raised as noted above in the subsection Two motivating examples, thus outweighing his higher sensitivity. We also observe that the overall magnitudes of the optimal shifts predicted for uniformly distributed coherences are substantially smaller, being 6.14% and 7.16% for A and T respectively, in comparison with 11.7% and 9.92% for the coherences used in the experiments.

**Figure 7:**
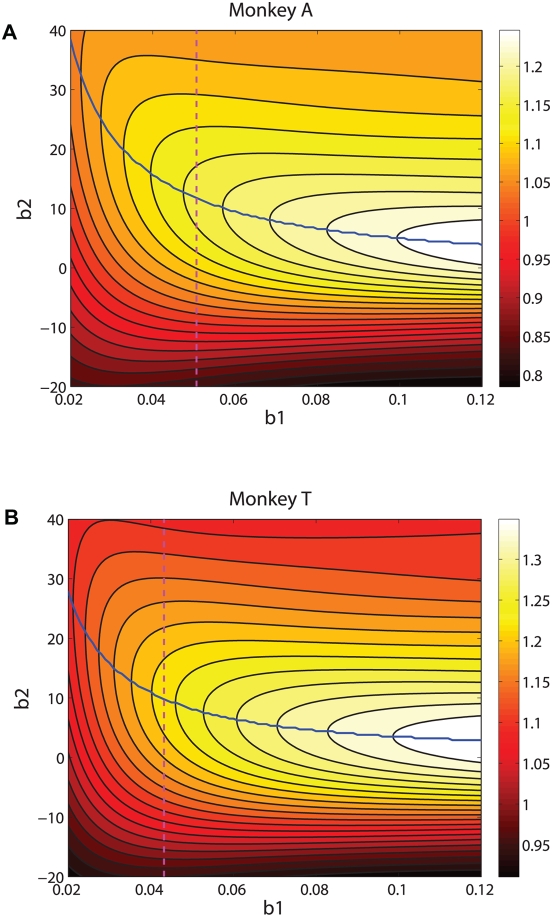
Contours (black curves) of expected rewards 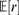 for 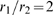 for monkeys A (A) and T (B) over the (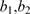)-plane, based on the coherences viewed by each animal. Vertical dashed lines indicate 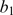 values fitted to pooled equal rewards data. Note that gradients in 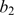 in either direction away from ridges of maximum expected rewards (blue curves) become smaller as 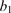 decreases, that gradients are smaller for overshifts in 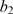 than for undershifts, that this asymmetry increases as 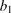 decreases, and that gradients are steeper for T than for A. See text for discussion.

While the overshifts for conditions HL and LH are significant in terms of coherence, it is important to assess how dearly they cost the animals in reduced rewards. In Figure 7 we plot expected reward functions (31) for 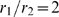 and the sets of coherences experienced by each animal (expected rewards for 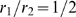 are obtained by reflecting about 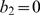). This reveals that, given the animals' averaged 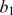 values (dashed magenta lines), the second derivatives 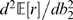 at the maxima are small, so the peaks are mild and deviations of ±10% coherence from 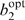 lead to reductions in expected rewards by only 2–3% from the maximum values (blue curves): an observation to which shall return below. Moreover, for unequal rewards the expected values decrease from their maxima more rapidly as 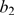 falls below 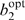 than they do for 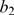 above 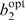. (The asymmetry becomes stronger as the reward ratio increases, and the curves are even functions when 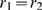 (not shown here).) This provides a rationale for the overshifting exhibited by the monkeys: smaller losses are incurred than in undershifting by the same amount. A similar observation appears in pp 728–729 of [8], in connection with the dependence of reward rate on decision threshold in a free response (reaction time) task.

**Figure 8:**
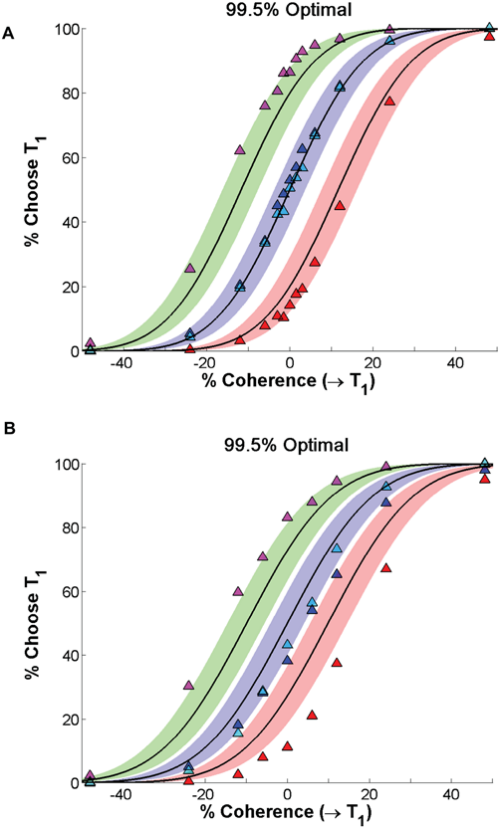
Optimal PMFs (black curves) and bands (color) in which 99.5% of maximal possible rewards are gained, compared with session-averaged HL, LL and HH, and LH data (triangles, left to right on each panel) for monkeys A (A) and T (B). See text for details.

**Figure 9:**
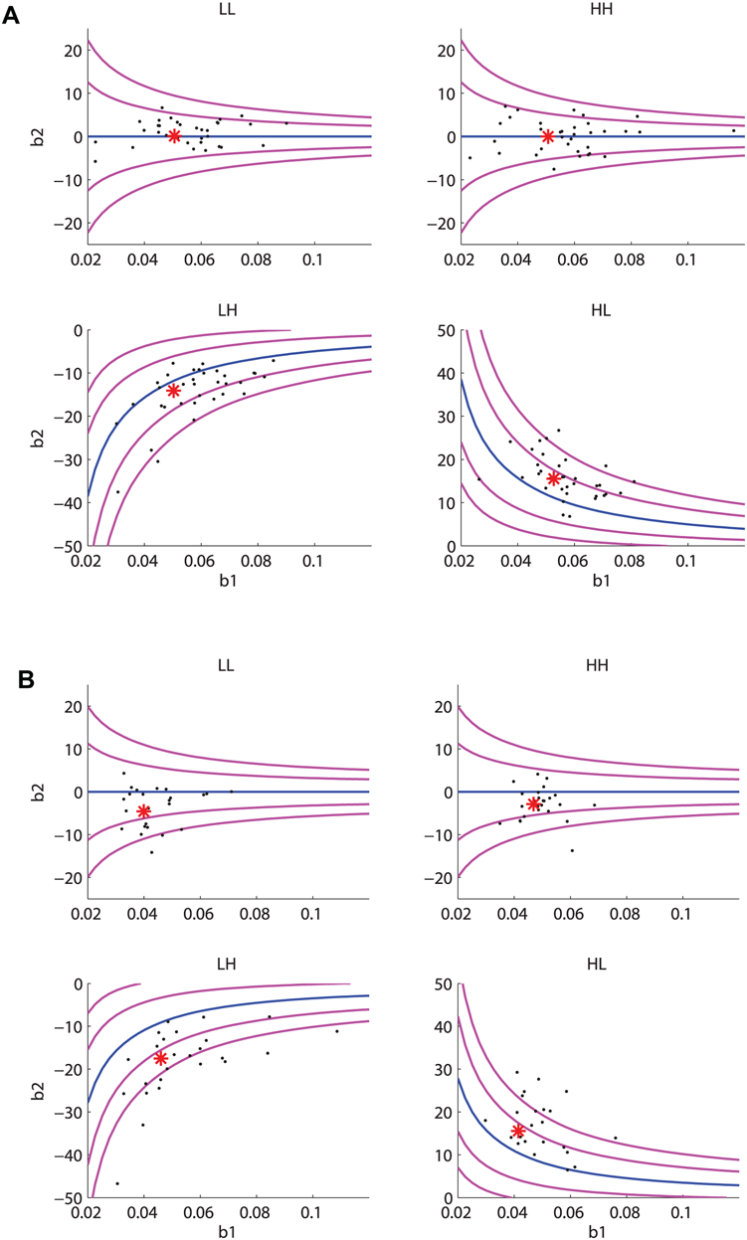
Slope and shift values for individual sessions and the four reward conditions, plotted as points in the 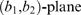 for monkeys A (four panels in (A)) and T (four panels in (B)). Asterisks indicate values averaged over all sessions (cf. top two rows of Table 1). Performance curves and bands show optimal 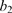 values for given 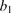 values (central blue curves) and values that gain 99% and 97% of maximum rewards are also shown (flanking magenta curves closest to and farthest from blue curves, respectively).

We conclude that, when averaged over all sessions, both animals' shifts err in the direction that is least damaging, and that neither suffers much penalty due to his overshift. Figure 8 further quantifies this by plotting the optimal PMF curves based on the slope values 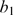 for pooled equal rewards (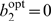), and with the symmetric optimal shifts 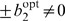 for the HL and LH reward conditions predicted by Eq. (33), along with bands that contain over- and under-shifted PMFs that garner 99.5% of the maximum rewards. With two exceptions (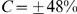), monkey A's mean shifts for all conditions lie within or on the borders of these bands. Monkey T is less accurate, exhibiting substantial shifts for the HH and LL conditions and significantly overshifting for unequal rewards (especially LH); even so, his rewards lie within 99% bands with the exception of that for the LH condition, which lies within the 98% band (not shown here, but see Figure 9 below).

#### Variability of behaviors in individual sessions

As Figures 5 and 8 illustrate, when averaged over all sessions, monkeys A and T respectively come within 0.5% (except for two outlying points) and 2% of achieving maximum possible rewards, given their limited sensitivities. However, the standard errors in Figure 5 show that their performances are quite variable. Indeed, the mean slopes 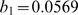 for A and 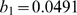 for T, obtained by averaging values fitted separately for each session, have standard deviations of 0.0116 and 0.0076 respectively (≈20% and 15% of their means). (These means differ from the averages of the four 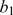 values in rows 1 and 2 of Table 1 because they were obtained by averaging the results of individual session fits, rather than from fits of data that was first averaged over sessions.)

Since both sensitivity, quantified by 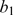, and shift (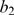) vary substantially from session to session, we asked if these parameters exhibit any significant correlations that would indicate that the animals are tracking the ridges of maxima on Figure 7. Specifically, from Eq. (33) we can compute values of 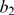 for which 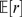 is maximized for given 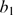 for reward ratios 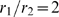 (HL) and 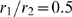 (LH), yielding loci of optimal shifts as a function of sensitivity, and from Eq. (31) we can deduce similar loci on which fixed percentages of maximum expected rewards are realised. In Figure 9 we compare the results of individual experimental sessions, plotted as points in the 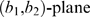, with these curves. The asterisks indicate the mean values of 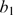 and 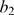 for each combination of animal and reward condition; the points indicate outcomes for individual sessions.

While in some cases the data seems to “parallel” the optimal performance contours (e.g., for both monkeys in condition LH and for A in conditions LL and HH), computations of Pearson's product moment correlation (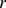) between 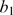 and 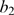 reveal weak correlations that approach or exceed 0.5 only if the unequally rewarded (HL and LH) data for each animal are pooled (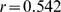, with a 95% confidence interval [0.351,0.689] for A; 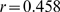 and [0.205,0.653] for T). Moreover, as noted by J. Gao and J. McClelland (personal communications), these parameters are not orthogonal. In the PMF of Eq. (15), 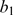 accounts for how coherence scales but it is the *product* 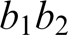 that describes the effect of unequal rewards: thus, a correlation between 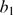 and 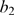 is to be expected.

Our optimality theory allows us to perform a more telling test. While we cannot extract an exact formula for the optimal covariation of 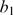 and 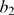 implicit in Eq. (33), Eq. (34) provides an excellent approximation for the blue curves of Figure 9, implying that individual session data should lie close to 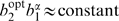 if the animals are tracking the ridges. Fitting values of 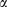 for A and T (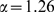 and 1.30 respectively) and comparing the HL and LH data sets with these curves gives considerably weaker correlations than those for 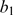 and 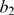 quoted above. We therefore conclude that no significantly-correlated adjustments of 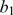 and 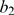 exist, and that random scatter dominates the individual session data.

## Discussion

We reduce a leaky competing accumulator model to an Ornstein-Uhlenbeck (OU) process, and therefrom derive a cumulative normal psychometric function (PMF) that describes how accuracy depends upon coherence (signal-to-noise ratio) in a two-alternative forced-choice task with cued responses. The key parameters in the PMF are its *slope* at 50% accuracy, which quantifies a subject's sensitivity to the stimulus, and its *shift*: the coherence at which 50% accuracy is realised. We compute analytical expressions describing optimal shifts that maximize expected rewards for given slopes and reward ratios. We find that this PMF can fit behavioral data from two monkeys performing a motion discrimination task remarkably well. The resulting slopes and shifts show that, faced with mixed coherences, while both animals “overshift” for unequal rewards, they nonetheless garner 98–99% of their maximum possible rewards (Figure 8), and they achieve this in spite of significant variability in sensitivity and shifts from session to session.

The linear OU process has the advantages of simplicity and it yields an explicit expression for the PMF, but it only approximates the dynamics of the decision process. Nonlinear drift-diffusion processes can also be derived from multi-dimensional models containing individual spiking neurons or neural pools [21],[41], but the Kolmogorov equations analogous to Eq. (6) cannot generally be solved and explicit expressions for PMFs are not available. Such more accurate models (with additional parameters) might provide better fits to data than the cumulative normal of Eq. (11), although the free response data presented in [41] indicates that there is little difference between linear and nonlinear models in fit quality per se. Nonlinear models do, however, better represent limiting neural behavior at high and low spike rates.

We also propose two simple methods by which the OU process could be biased by reward expectations, in order to produce such shifts. The first requires a biased starting point for evidence accumulation, the second assumes a continuing bias to the drift rate that enters the OU process prior to and throughout the stimulus viewing period. In the free response case, with blocked trials and fixed coherence in each block, it is known that the former is optimal [8], and recent experiments focusing on stimulus proportions confirm that well-practiced human subjects do approximate this [49]. As described under Models of stimuli and reward biasing, the fixed viewing time experiment employed here cannot distinguish among these or other biasing models. Responses gathered for different reward cue and motion periods would enable such distinctions; cf. [25]. Accumulator models have also been proposed for working memory following stimulus offset (e.g. see [50] for a somatosensory comparison task). Addition of such a model and analysis of electrophysiological data throughout the trial, including the variable delay period, may further illuminate the biasing mechanism.

Our optimality analysis presumes that the PMF slope (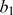) has an upper bound that reflects fundamental limits on sensitivity to the visual stimulus. We then seek the unique shift (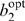) that maximizes expected rewards over the given coherence and reward conditions, for a fixed slope. This makes for a well-posed mathematical analysis, but it does not imply that the animal is faced with a given sensitivity and then “chooses” a shift. He might equally well choose a shift and then “accept” a sensitivity that delivers adequate rewards, perhaps by implicitly selecting a weight for the top-down reward information, and then relaxing attention to the stimuli until his reward rate reaches a predetermined level. He may even co-vary these parameters to achieve the same end. This is reminiscent of a robust-satisficing strategy that has been studied in connection with setting speed-accuracy tradeoffs [51].

A related study of optimal decision strategies in two-alternative forced-choice tasks with free responses has shown that decision thresholds can be determined for a pure drift diffusion process that optimize reward rate by setting a speed-accuracy tradeoff [8]. In that work it is necessary to assume that trials are blocked (e.g. with equal coherences 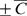), so that conditions remain statistically stationary during each session and one can appeal to optimality of the DD process [43]. In contrast, for cued responses only the accuracy level need be maximized, one need not assume a pure DD process, and optimization can be done in the face of mixed coherences and mixed reward contingencies. As the theory developed above shows, reduction to a one-dimensional process permits explicit calculations of PMFs and optimality conditions, and comparison with data requires only simple two parameter fits. However, the present behavioral data lacks the reaction time distributions that allow fits that could distinguish among multiparamater variants of DD and OU models [15],[22],[52],[53].

We have taken as a utility function 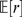 the (normalised) value of expected rewards, implicitly assuming that two drops of juice are worth twice one drop. Subjective utility may not vary linearly with reward size: for example, at high reward ratios it may rise more slowly and saturate due to satiety. In contrast, if we suppose that two drops of juice are worth 2.5 or 3 times as much as one drop, then the shifts of both animals would lie much closer to the optimal curves of Figure 6 (translate the HL data points horizontally from 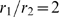 to 2.5 or 3, and the LH data points from 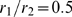 to 0.4 or 0.33). However, a study of subjective value quantification would require investigation of a broad range of reward ratios.

The behavioral data analyzed here were obtained simultaneously with electrophysiological recordings from single neurons in the lateral intraparietal area (LIP) of the cerebral cortex, a region that is thought to play a key role in the formation of oculomotor decisions within the central nervous system [7],[19],[34]. The results presented in this paper raise important questions for our ongoing analysis of the neurophysiological data. Do decision-related neurons in LIP encode or at least reflect effects of both the reward prior and the coherence of the visual stimuli? Are the two effects present in the same proportions at the neural level as at the behavioral level (as quantified in the present paper)? Is the effect of reward bias evident as an offset at the start of accumulation of motion information by LIP neurons, or as a gain factor on the accumulation process, or both? These questions will be addressed in a future publication integrating neurophysiological data with the behavioral results.
